# ZIP8 expression in human proximal tubule cells, human urothelial cells transformed by Cd^+2^ and As^+3^ and in specimens of normal human urothelium and urothelial cancer

**DOI:** 10.1186/1475-2867-12-16

**Published:** 2012-07-04

**Authors:** Amornpan Ajjimaporn, Tom Botsford, Scott H Garrett, Mary Ann Sens, Xu Dong Zhou, Jane R Dunlevy, Donald A Sens, Seema Somji

**Affiliations:** 1 Department of Pathology, School of Medicine and Health Sciences, University of North Dakota, Grand Forks, ND, USA; 2 Anatomy and Cell Biology, School of Medicine and Health Sciences, University of North Dakota, Grand Forks, ND, USA; 3 Department of Pathology, School of Medicine and Health Sciences, University of North Dakota, 501 N. Columbia Road, Grand Forks, ND, 58202, USA

**Keywords:** Zinc transport, Cadmium transport, ZIP8, Proximal tubule, Renal toxicity, Urothelium, Urothelial cancer, Arsenic

## Abstract

**Background:**

ZIP8 functions endogenously as a Zn^+2^/HCO_3_^-^ symporter that can also bring cadmium (Cd^+2^) into the cell. It has also been proposed that ZIP8 participates in Cd-induced testicular necrosis and renal disease. In this study real-time PCR, western analysis, immunostaining and fluorescent localization were used to define the expression of ZIP8 in human kidney, cultured human proximal tubule (HPT) cells, normal and malignant human urothelium and Cd^+2^ and arsenite (As^+3^) transformed urothelial cells.

**Results:**

It was shown that in the renal system both the non-glycosylated and glycosylated form of ZIP8 was expressed in the proximal tubule cells with localization of ZIP8 to the cytoplasm and cell membrane; findings in line with previous studies on ZIP8. The studies in the bladder were the first to show that ZIP8 was expressed in normal urothelium and that ZIP8 could be localized to the paranuclear region. Studies in the UROtsa cell line confirmed a paranuclear localization of ZIP8, however addition of growth medium to the cells increased the expression of the protein in the UROtsa cells. In archival human samples of the normal urothelium, the expression of ZIP8 was variable in intensity whereas in urothelial cancers ZIP8 was expressed in 13 of 14 samples, with one high grade invasive urothelial cancer showing no expression. The expression of ZIP8 was similar in the Cd^+2^ and As^+3^ transformed UROtsa cell lines and their tumor transplants.

**Conclusion:**

This is the first study which shows that ZIP8 is expressed in the normal urothelium and in bladder cancer. In addition the normal UROtsa cell line and its transformed counterparts show similar expression of ZIP8 compared to the normal urothelium and the urothelial cancers suggesting that the UROtsa cell line could serve as a model system to study the expression of ZIP8 in bladder disease.

## Background

Cadmium is ranked 7^th^ in the “Top 20 Hazardous Substances Priority List “ by the Agency for Toxic Substance and Disease Registry and the U.S. Environmental Protection Agency [1]. Individuals at the highest risk for cadmium-related disease include cigarette smokers, those on a steady diet rich in high fiber foods or contaminated shellfish, women having low body-iron stores, and malnourished populations [2-5]. In acute doses, Cd^+2^ has been shown to cause damage to the central nervous system, lung, bone, gastrointestinal tract, liver, ovary, testis, placenta, and the developing embryo [6,7]. Chronic exposure to low amounts of Cd^+2^ has been shown to cause renal proximal tubular metabolic acidosis and osteomalacia (renal Fanconi syndrome) [8]. The elimination of Cd^+2^ from the body is very slow and thus accumulates as a total body burden, predominantly in the kidney, with age. Cadmium is also classified as a human “Category 1” carcinogen due to its strong correlation with lung cancer [5,9-11]. Association of Cd^+2^ with cancers of other organs have also been suggested, but the data are currently inconclusive. This laboratory has been interested in the possible association of Cd^+2^ with the development and progression of human urothelial cancer. There is an extremely strong association of human bladder cancer with the consumption of cigarettes and tobacco, with some reports suggesting a two- to four-fold increased risk and that 50% of the bladder cancers in men would not occur in the absence of cigarette smoking [12,13]. The number of cigarettes smoked, degree of inhalation, type of tobacco, use of filters, and smoking cessation have all been shown to have specific relationships with the development of bladder cancer [14]. Cigarette smoke is by far one of the greatest sources of Cd^+2^ exposure with each cigarette containing between 1–2 μg of Cd^+2^ and 40-60% of the Cd^+2^ in inhaled smoke enters the systemic circulation [7,13-17]. The high level of Cd^+2^ accumulation in individuals who smoke cigarettes, along with the strong association of bladder cancer and smoking, is a major factor indirectly implicating Cd^+2^ in the development of urothelial cancer. There are also several epidemiological studies which have implicated Cd^+2^ in the development of bladder cancer [5,9,18,19].

This laboratory has developed a model of Cd^+2^ and As^+3^ induced human urothelial cancer through the direct malignant transformation of the UROtsa cell line, a cell line retaining characteristics of human urothelium [20,21]. This laboratory has also been interested in As^+3^, a bladder carcinogen highly implicated in the incidence of bladder cancers due to human exposure in drinking water [22-24], and have developed an *in vitro* bladder carcinogenesis model for As^+3^ similar to that of Cd^+2^. The As^+3^-transformed cells serve as an interesting control to that transformed by Cd^+2^, due to the divergent chemical properties of As^+3^. The laboratory has subsequently isolated and characterized 6 additional Cd^+2^ transformed cell lines and 5 additional As^+3^ transformed cell lines [25-27]. These cell lines were all shown to retain morphological characteristics consistent with human urothelial cancer and to display phenotypic differences characteristic of tumor heterogeneity. The histology of subcutaneous tumor transplants produced by these transformed isolates displayed histological features of human urothelial carcinoma with areas of squamous differentiation. This observation is important since urothelial carcinoma is the most prominent type of bladder cancer in western countries and accounts for over 95% of all cases and is 5^th^ in overall occurrence [28]. To the authors’ knowledge, there has been no examination of the mechanism by which Cd^+2^ might enter the urothelial cell in order to elicit cell transformation. Recent studies have shown a relationship between a specific allelic difference in the mouse *Slc39a8* gene encoding the ZIP8 transporter and the specific phenotypes of Cd-induced testicular necrosis and acute renal failure [29,30]. Subsequent studies have shown that the ZIP8 transporter, which is utilized by Cd^+2^ for transport can also transport one or more essential divalent cation(s) that are critical to cellular function [31]. In cell culture studies, manganese (Mn) was shown to be the best inhibitor of ZIP8-mediated Cd^+2^ uptake; possessing a low Km of 2.2 μM. These studies show that ZIP8 is a Cd^+2^ or Mn^+2^/HCO_3_^-^ symporter, but a role for the transport of Zn^+2^ cannot be ruled out. ZIP8 has been localized to the apical surface of two cell types; between the blood and vascular endothelial cells of the testis [29,30], and between the glomerular filtrate and renal proximal tubule cells [30]. ZIP8 has also been shown to exist in glycosylated and non-glycosylated forms [30,31] and can alter their localization as a function of extracellular Zn^+2^ concentration [32]. The role of ZIP transporters in cadmium damage to the testis and kidney has been the subject of a recent review [33]. The finding that the ZIP8 transporter can transport Cd^+2^ into several cell types suggested that this transporter might also be operative in the urothelial cell. The first goal of the present study was to determine the expression and localization of ZIP8 in HPT cells since the *in situ* expression of ZIP8 has previously been shown for this cell type. The second goal was to determine if ZIP8 was expressed in normal human urothelium and if expression was altered in human urothelial cancer. The final goal of the study was to determine ZIP8 expression and localization in human urothelial cells transformed by Cd^+2^ and As^+3^.

## Results

### Expression and localization of ZIP8 in human kidney and cultured HPT cells

**Figure 1:**
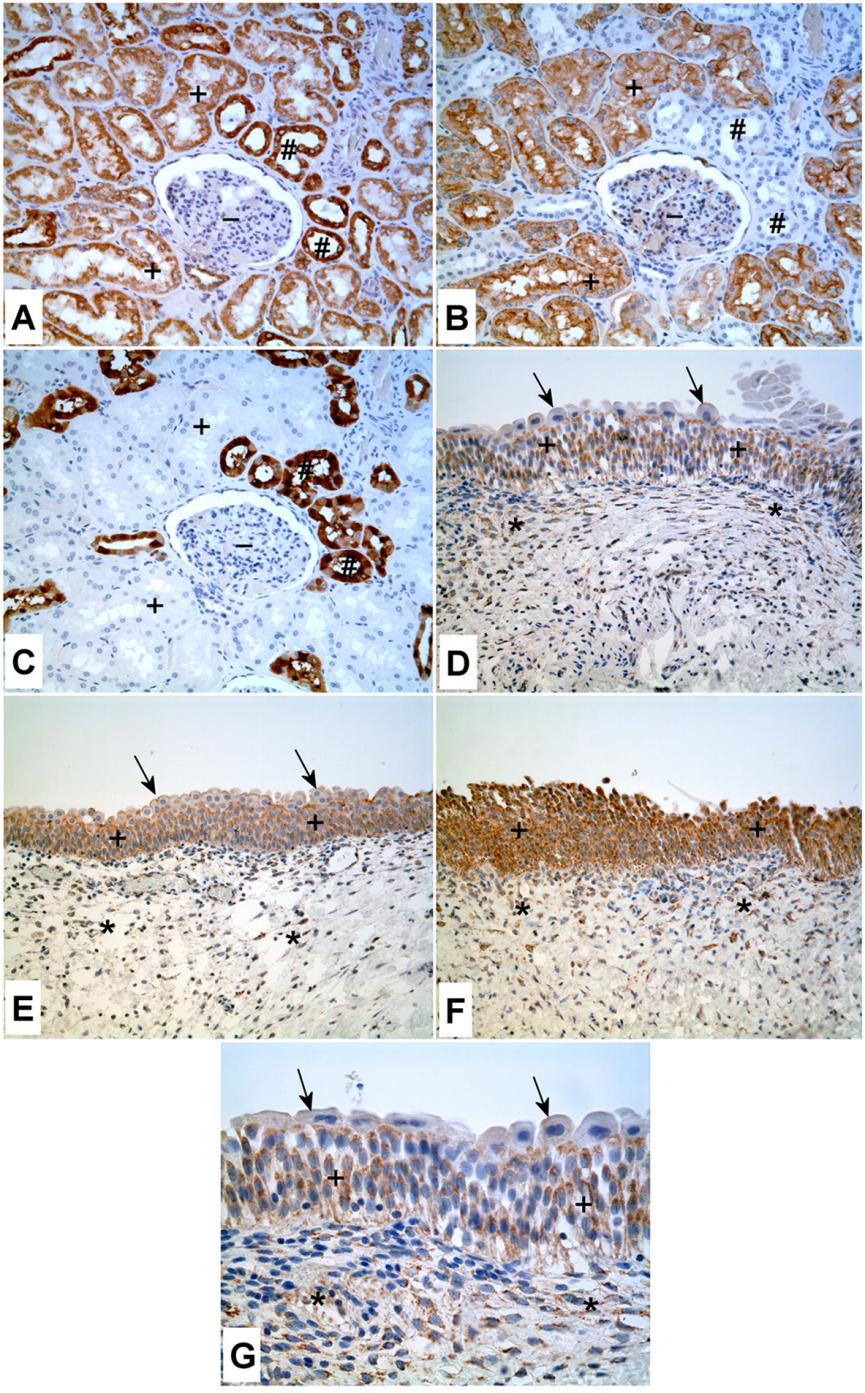
**Immunoperoxidase staining of ZIP8 in normal kidney and urothelium.A.** Normal kidney. Staining of ZIP8 in the distal tubule (#) is stronger compared to the proximal tubules (+), whereas the staining is absent in the glomerulus (−). **B** and **C.** Serial section of the normal kidney stained for aquaporin-1 **(B)**, a proximal tubule specific marker and calbindin **(C)**, a distal tubule specific marker. (+) indicates proximal tubules and (#) indicates distal tubules. **D-G.** Staining of ZIP8 in the normal human urothelium. **D.** The urothelium from bladder shows weak staining of ZIP8 (+), mainly in the intermediate layers; the most superficial umbrella cells are negative for ZIP8 (arrows). Some spindled stromal cells in lamina propria are weakly positive for ZIP8 (*). **E.** The urothelium from renal pelvis shows moderate staining of ZIP8 in the intermediate layers (+); the staining of the most superficial umbrella cells (arrows) is much weaker than the intermediate layers. The asterisks indicate stromal tissue in lamina propria, which shows no staining of ZIP8. **F.** Strong staining of ZIP8 in the urothelium (+). Some scattered stromal cells in lamina propria are also positive for ZIP8 (*). **G.** Higher magnification of **D**, displaying paranuclear staining of ZIP8 in the cells of intermediate layers of urothelium (+). The umbrella cells are almost negative for ZIP8 (arrows). Asterisks mark the stromal cells in lamina propria with weak staining of ZIP8.

**Figure 2:**
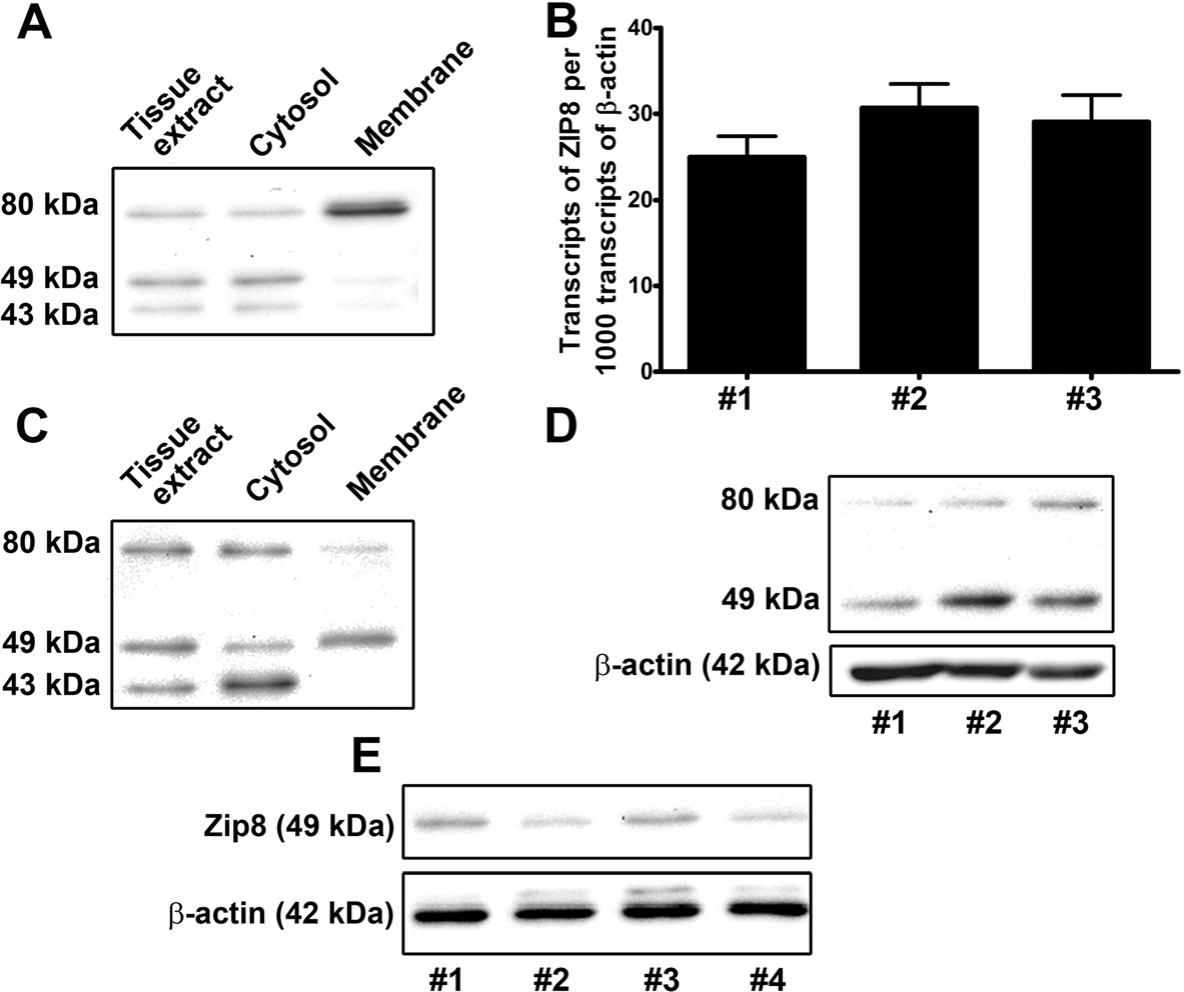
**Expression of ZIP8 in the human kidney and bladder.A.** Western analysis of ZIP8 protein in cortical tissue of the human kidney. **B** and **D.** Expression of ZIP8 in cultured HPT cells. **B.** Real-time PCR analysis of ZIP8 mRNA. **D.** Western analysis of ZIP8 protein. **C.** Western analysis of ZIP8 protein in cultured UROtsa parent cell. **E.** Western analysis of ZIP8 protein in normal human urothelium tissue.

**Figure 3:**
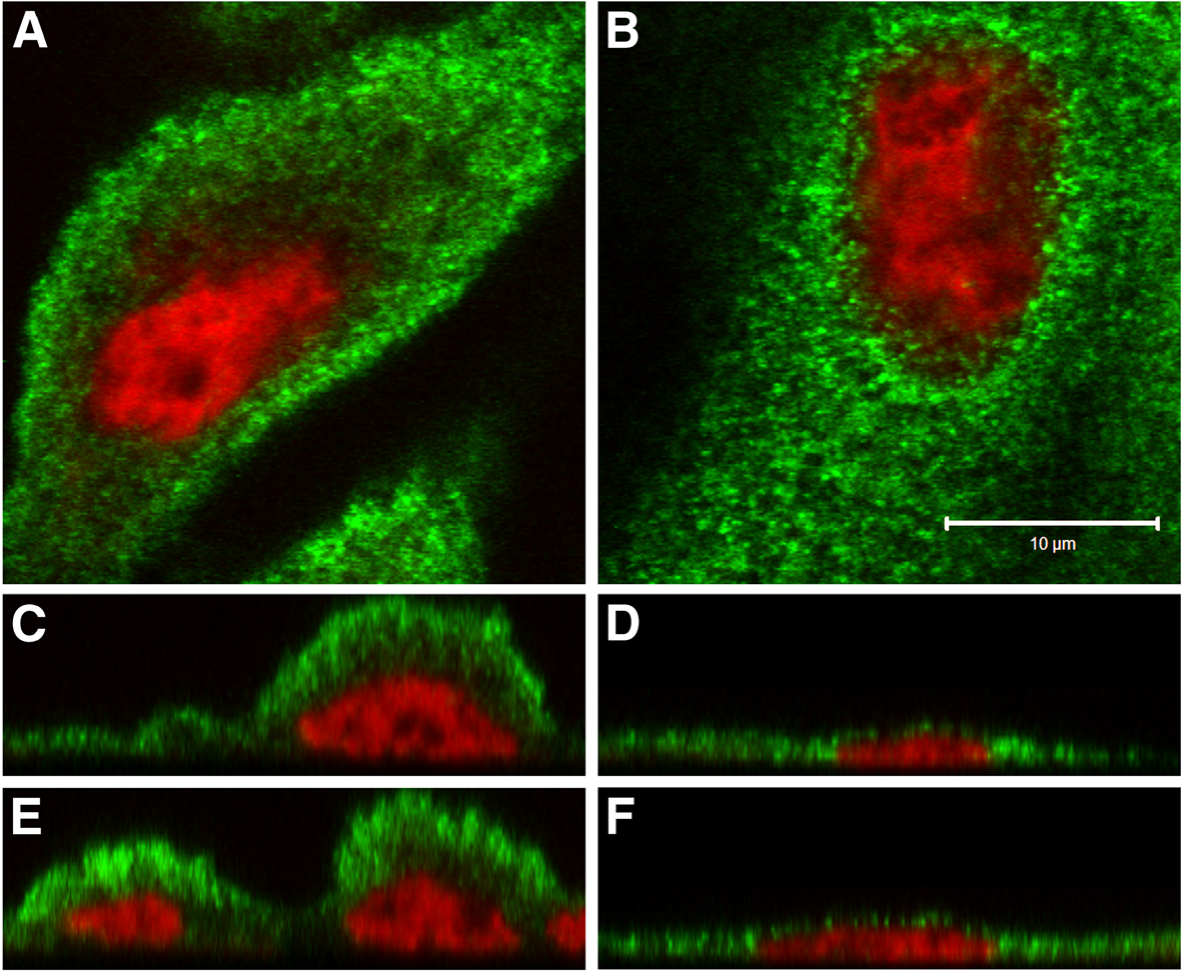
**Immunofluorescent localization of ZIP8 in HPT cells.** The two patterns of localization are shown. The first is a concentration of ZIP8 at the apical face **(A, C, E)** which is the dominant localization observed. The second is a concentration of ZIP8 in the paranuclear region **(B, D, F).** All HPT cells showed ZIP8 extending throughout the cytoplasm in a punctate pattern consistent with the endoplasmic reticulum. ZIP8 staining is shown in green while the nuclear dye To-PRO-3 iodide is shown in red. Single Z-series slices are shown in **A** and **B** while orthogonal views in the X- and Y-planes are shown in **C**, **D** and **E**, **F** respectively. Bar = 10 μm and represents the scale for both **A** and **B.**

The ZIP8 protein has been previously reported to be expressed in the proximal tubule of the mouse kidney and to exist in glycosylated and non-glycosylated forms [30,31]. In the present analysis, this observation was extended to the human kidney and cultured HPT cells. Immunohistochemisty was used to determine the expression and localization of ZIP8 protein in paraffin-embedded, formalin-fixed, patient archival specimens. Serial sections of the kidney specimens were cut and stained for ZIP8, aquaporin-1 and calbindin. Three independent specimens of human kidney were examined and all demonstrated expression of ZIP8 in the proximal and distal tubules (Figure 1A). The illustration shown is typical of all 3 independent samples which showed strong staining of the distal tubules and moderate to strong staining of the proximal tubules. Glomeruli were negative for ZIP8 staining in all samples which served as an additional negative control, over that of staining with primary and secondary antibody alone. Staining for aquaporin-1 (proximal tubule specific marker) (Figure 1B) and calbindin (distal tubule specific marker) (Figure 1C) confirmed the identification of the different tubules. Western analysis was performed on protein extracts prepared from the three samples of human renal cortex. The results of this analysis showed that whole cell extract of normal renal cortex displayed three bands reactive with the ZIP8 antibody (Figure 2A). The three ZIP8 protein bands had molecular weights corresponding to approximately 80, 49 and 43 kDa. Real-time PCR and western analysis was also performed on extracts of 3 isolates of HPT cells. The expression of ZIP8 mRNA was similar among all three cell isolates and well below that of the β-actin gene, having about 25 ZIP8 transcripts for every 1,000 transcripts of β-actin (Figure 2B). Western analysis (Figure 2D) showed the presence of two bands of ZIP8, one corresponding to a molecular weight of 80 kDa and the other 49 kDa. The expression of the 49 kDa band was much higher than that of the 80 kDa ZIP8 band. In contrast to that found in renal tissue, there was no evidence of the 43 kDa ZIP8 protein band in extracts of the HPT cells. Immunofluorescence confocal microscopy was used to localize the ZIP8 protein in the HPT cells. The results of this analysis showed that the ZIP8 protein was localized at two distinct cellular locations within the population of HPT cells (Figure 3). The first, which was the dominant pattern, showed localization of the ZIP8 protein in a punctate pattern that extended throughout the cytoplasm of the cell with an increased concentration along the periphery of the apical face of the cell (Figure 3A, C, E). The punctate cytoplasmic localization is consistent with presence in the endoplasmic reticulum (ER) and the peripheral staining indicates localization to the cell membrane. The second pattern noted in fewer cells showed a similar localization of the ZIP8 protein throughout the cytoplasm consistent with localization to the ER. However, in these cell profiles, a distinct concentration of ZIP8 staining was localized to the paranuclear region of the cell and not to the periphery of the cell (Figure 3B, D, F).

### Expression and localization of ZIP8 in human urothelium, urothelial cancer and parental UROtsa cells

Immunohistochemisty was also used to determine the expression and localization of ZIP8 protein in paraffin-embedded, formalin-fixed, patient archival specimens of normal human urothelium and urothelial cancer. Five independent specimens of normal urothelium were examined for the expression and localization of the ZIP8 protein. The specimens of “normal” urothelium were archival specimens from patients undergoing surgical intervention for bladder cancer and showed no cancer involvement on hematoxylin and eosin (H&E) examination of the tissue sections. The ZIP8 protein was shown to be expressed in the urothelial cells of all five specimens of normal bladder; however, the expression in the urothelial cells was variable among the specimens (Figure 1D, E, F). One specimen was shown to have weak expression of ZIP8 in the urothelium (Figure 1D), three specimens had moderate expression (Figure 1E) and one specimen showed strong staining for ZIP8 (Figure 1F). Within each specimen, ZIP8 staining of the urothelial cells was uniform among the cells. There were occasional spindle cells in the lamina propria that showed weak ZIP8 staining, but otherwise the majority of stromal cells showed no staining for ZIP8. The immunohistochemical analysis of ZIP8 was also informative regarding the localization of ZIP8 in normal urothelium. Low power microscopic examination suggested, in addition to localization in the cytoplasm, a paranuclear localization of ZIP8 staining in the urothelial cells and this was confirmed by examination at higher powers of light level microscopic examination (Figure 1G). This finding was consistent for all five specimens of normal urothelium. Western analysis was used to determine the expression of the ZIP8 protein in extracts prepared from 4 independent samples of normal human urothelium. These samples were obtained as medical waste without patient identifiers from surgically removed bladder cancer specimens following completion of diagnostic protocols in surgical pathology. The specimens were selected by the attending pathologist to be areas of the urothelium removed from those areas of the bladder having urothelial cancer. This analysis showed that all 4 specimens displayed the 49 kDa band identified as the non-glycosylated form of the ZIP8 protein (Figure 2E). None of the 4 samples of normal urothelium showed the presence of the higher molecular weight 80 kDa protein band associated with the glycosylated form of ZIP8 (Figure 2E). Cytosolic and membrane extracts were also prepared from parental UROtsa cells and western analysis was performed on the protein samples. The analysis showed that all the three forms of ZIP8 protein (80, 49 and 43 kDa) were present in the whole cell extract as well as the cytosolic extracts (Figure 2C). The 80 and the 49 kDa bands were found to be associated with the membrane preparation.

**Figure 4:**
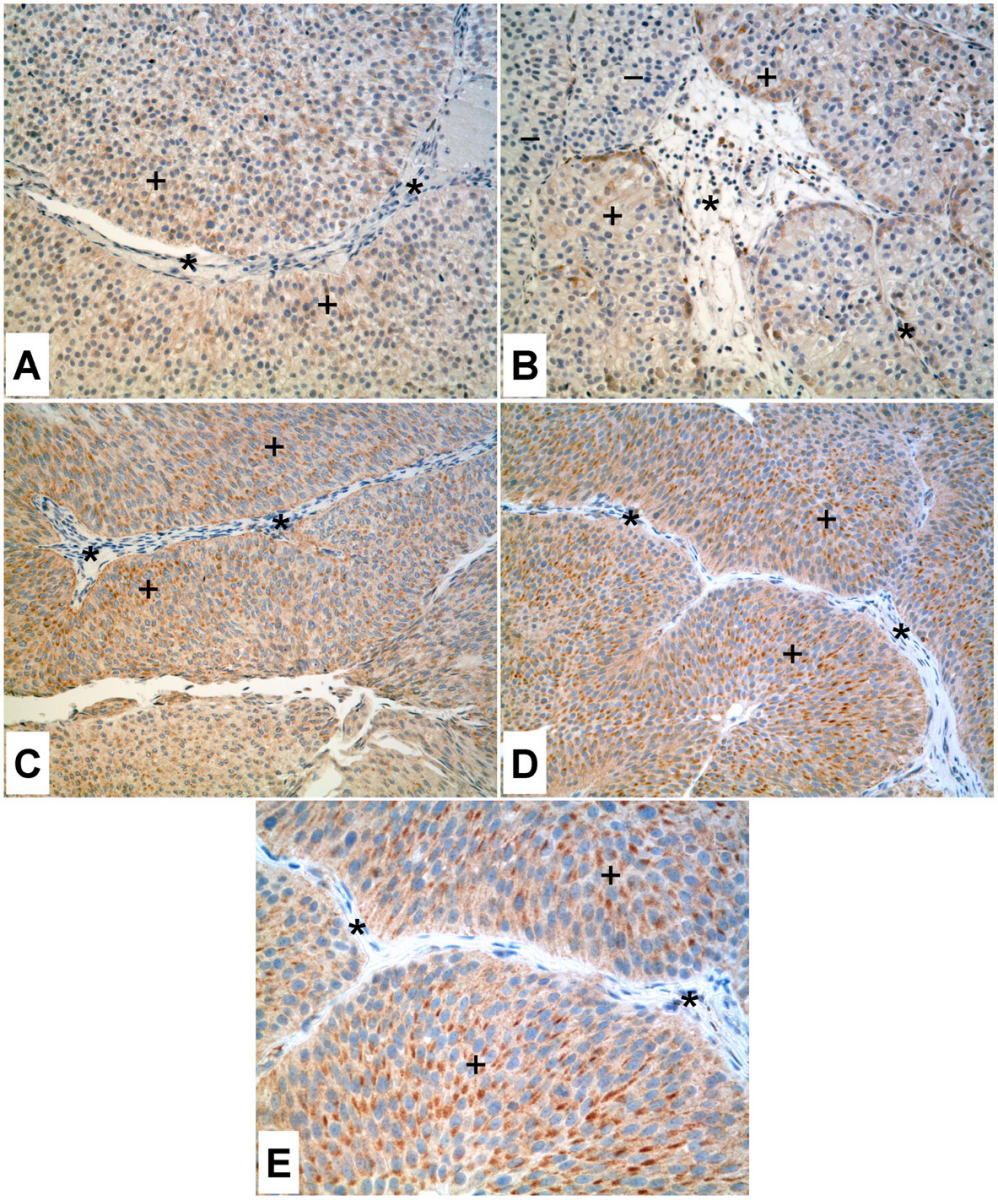
**Immunoperoxidase staining of ZIP8 in low grade urothelial carcinoma.A.** Weak staining of ZIP8 (+) in low grade urothelial carcinoma. Asterisks (*) indicate stromal tissue which is negative for ZIP8. **B.** low grade urothelial carcinoma of bladder showing weak focal staining of ZIP8 (+). There are areas of the tumor that show negative staining for ZIP8 (−). Asterisks (*) indicate stromal tissue which is negative for ZIP8. **C.** Low grade urothelial carcinoma from renal pelvis with moderate staining of ZIP8 (+). The tumor stroma is negative for ZIP8 (*). **D.** Moderate staining of ZIP8 (+) in low grade urothelial carcinoma. Asterisks (*) indicate stromal tissue which is negative for ZIP8. **E.** Higher magnification of **D** showing paranuclear staining of ZIP8 (+). Asterisks (*) indicate stromal tissue which is negative for ZIP8.

**Figure 5:**
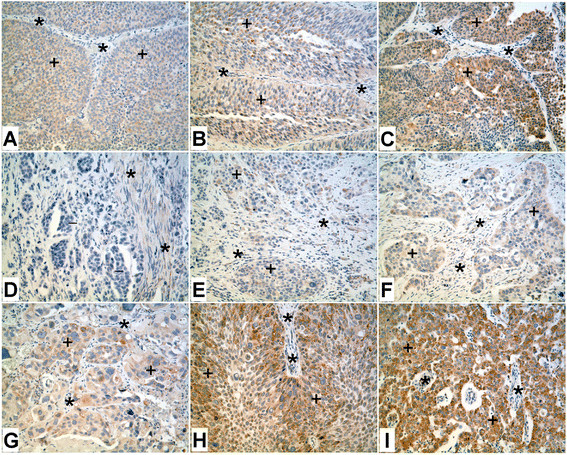
**Immunoperoxidase staining of ZIP8 in high grade urothelial carcinoma.A** &**B.** Noninvasive urothelial carcinoma showing weak staining of ZIP8 (+). Asterisks (*) indicate stromal tissue which is negative for ZIP8. **C.** Noninvasive urothelial carcinoma with focal moderate staining of ZIP8 (+). Asterisks (*) indicate stromal tissue which is negative for ZIP8. **D.** Invasive urothelial carcinoma with negative staining of ZIP8 (−). Few smooth muscle fibers between the invasive tumor nests are weakly positive for ZIP8 (*). **E.** Invasive urothelial carcinoma with focal weak staining of ZIP8 (+). Asterisks (*) indicate stromal tissue which is negative for ZIP8. **F.** Invasive urothelial carcinoma with weak staining of ZIP8 (+). A few spindled shaped stromal cells are also weakly positive for ZIP8 (*). **G.** Anaplastic urothelial carcinoma showing moderate staining for ZIP8 (+). Asterisks (*) indicate stromal tissue which is negative for ZIP8. **H.** Invasive urothelial carcinoma with moderate to strong staining of ZIP8 (*). A few spindled shaped stromal cells are weakly positive for ZIP8 (*). **I.** Poorly differentiated urothelial carcinoma with strong staining for ZIP8 (+). Asterisks (*) indicate stromal tissue which is negative for ZIP8.

Immunohistochemistry was also employed to examine the expression of ZIP8 in a small set of urothelial cancers. The expression and localization of ZIP8 was determined in 4 specimens of low grade urothelial cancer. All of the specimens displayed diffuse weak to moderate staining for ZIP8 (Figure 4A-D) and they also displayed moderate paranuclear staining for ZIP8 (Figure 4E). The stromal elements of the 4 specimens were uniformly negative for expression of ZIP8. Nine cases of high grade urothelial cancer were also examined for ZIP8 expression. Three cases were high grade non-invasive urothelial carcinomas and two of them displayed uniform diffuse weak staining of ZIP8 while the other displayed moderate, but focal, diffuse cytoplasmic staining for ZIP8 (Figure 5A, B, C). The stromal cells in each of these 3 cases of high grade, non-invasive urothelial cancer did not stain for ZIP8. Six cases of high grade, invasive urothelial cancer were examined for the expression of ZIP8 (Figure 5D-I). The expression of ZIP8 among these six cases covered a spectrum of expression, with one case having no expression (Figure 5D), two cases showing weak staining (Figure 5E, F), one case with moderate to strong staining (Figure 5G), and two cases with strong staining (Figure 5H, I). The expression was diffuse within the cytoplasm of all these cases. The tumor stroma in these six cases was mostly negative for ZIP8 expression, but an occasional stromal cell could be found that was weakly positive. None of the cases of high grade urothelial cancer displayed paranuclear staining of ZIP8.

### Expression and localization of ZIP8 in parental and Cd^+2^ and As^+3^ transformed UROtsa cells

**Figure 6:**
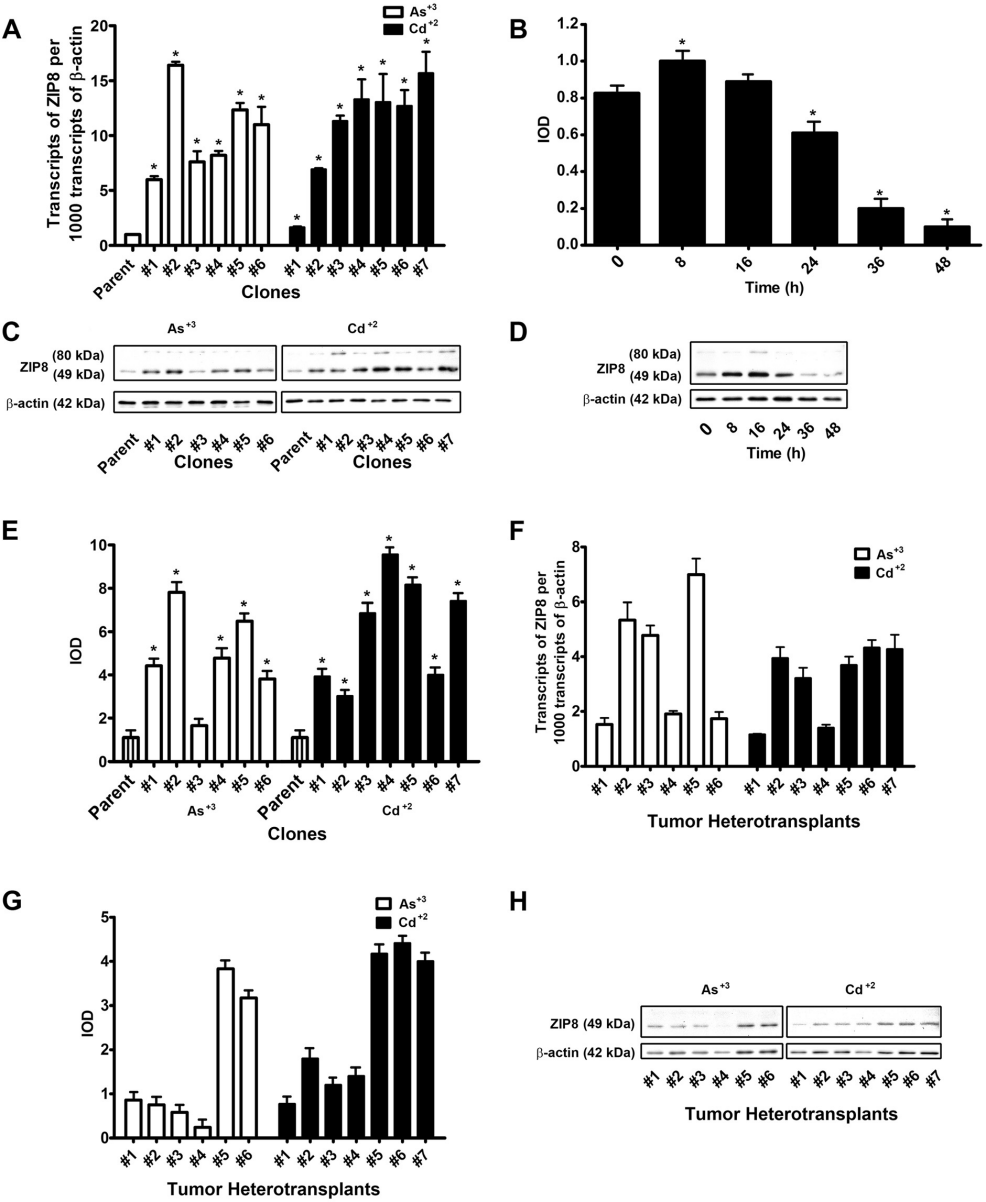
**Expression of ZIP8 in UROtsa cell lines transformed by As**^**+3**^**and Cd**^**+2**^**and their corresponding tumor heterotransplants.A.** Real-time PCR analysis of ZIP8 mRNA in As^+3^ and Cd^+2^ transformed cell lines. The analysis was done in triplicates and the results are expressed as mean transcript number per 1000 transcripts of β-actin. * Statistically significant compared to parental UROtsa cells. **B** and **D.** Effect of feeding on the expression level of ZIP8 protein in UROtsa parent cells. **B.** Mean (+) IOD values are for lower bands representing the 49 kDa form of ZIP8. **C** and **E.** Western analysis of ZIP8 protein in UROtsa cells transformed by As^+3^ and Cd^+2^. Mean (+) IOD values are for lower bands **(D)** representing the 49 kDa form of ZIP8. **F,G** and **H.** Expression of ZIP8 in tumor heterotransplants. **F.** Real-time PCR analysis of ZIP8 mRNA. **G** and **H.** Western analysis of ZIP8 protein. Mean (+) IOD values are for lower bands representing the 49 kDa form of ZIP8.

Real-time PCR was employed to determine the expression of ZIP8 mRNA in the parental UROtsa cell line and in the 6 As^+3^ and 7 Cd^+2^ transformed cell lines (Figure 6A). This analysis showed that expression of ZIP8 mRNA in the parental UROtsa cell line was on the order of 1 transcript for every 1,000 transcripts of β-actin mRNA. The expression of ZIP8 mRNA was elevated between 7 and 17 folds compared to the parental cells in all the cell lines transformed by As^+3^ or Cd^+2^. Western analysis was employed to determine the level of ZIP8 expression in the parental and As^+3^ and Cd^+2^ transformed cell lines. Preliminary determinations showed a wide variability in the expression of the ZIP8 protein in the parental UROtsa cells. To explore this variability, ZIP8 protein was determined by western analysis on parental cultures of UROtsa cells at 8, 16, 24, 36 and 48 hours (hrs) following the addition of fresh growth media. The results of this analysis demonstrated that the expression of the 49 kDa ZIP8 protein in the parental UROtsa cells was increased markedly 8 hrs and 16 hrs following the addition of fresh growth media to the cells, with a return to near pre-feeding levels by 24 hrs post-feeding (Figure 6B, D). A minor band consistent with the 80 kDa protein could be seen 16 hrs following addition of fresh growth media. An identical analysis on the transformed lines showed that ZIP8 mRNA expression was unaffected by the change in growth medium, remaining at levels not significantly different from that shown in panel A (Figure 6A). The 7 isolates of Cd^+2^ transformed UROtsa cells and the 6 As^+3^ transformed isolates were found to have no alterations in ZIP8 protein expression following replenishment of the growth media (data not shown). The expression of ZIP8 protein was determined in the 7 isolates of Cd^+2^ transformed UROtsa cells and the 6 As^+3^ transformed cell lines (Figure 6C, E). All the isolates were shown to express both the 49 kDa and 80 kDa protein bands, with the 49 kDa band being the most prominent. The expression of ZIP8 in the transformed isolates was compared relative to the parental UROtsa cells 24 hrs following replenishment of the growth medium. Using this time point for comparison, the data shows all but one isolate (As#3) to have increased expression of the 49 kDa ZIP8 protein.

**Figure 7:**
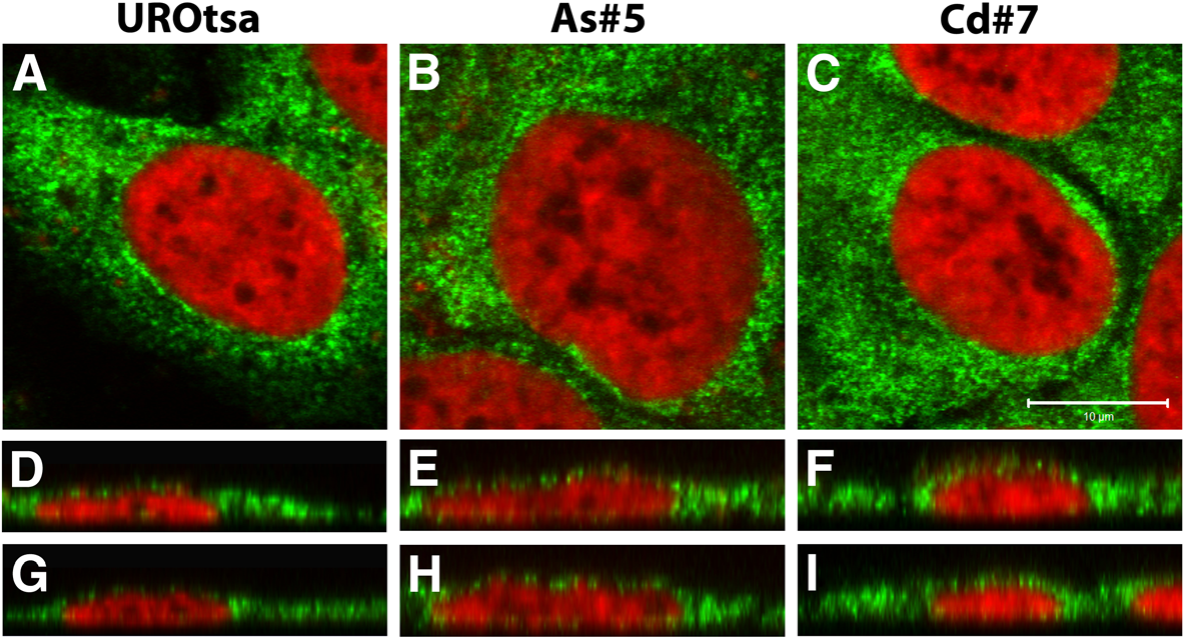
**Immunofluorescent localization of ZIP8 in UROtsa parent and transformed cell lines.** ZIP8 was found to concentrate heavily in the paranuclear region of the cells along with a punctate staining that extended throughout the cytoplasm, indicative of association with the endoplasmic reticulum. The parent UROtsa cells are shown in **A,D,G** while the transformed cells As#5 are shown in **B**, **E**, **H** and Cd#7 are shown in **C**, **F**, **I.** ZIP8 staining is shown in green while the nuclear dye To-PRO-3 iodide is shown in red. Single Z-series slices are shown in **A** - **C** while orthogonal views in the X- and Y-planes are shown in **D-F** and **G-I** respectively. Bar = 10 μm and represents the scale for **A** - **C.**

The intracellular localization of the ZIP protein was determined by immunofluorescent analysis and confocal microscopy in the parental UROtsa cells and their Cd^+2^ and As^+3^ transformed counterparts. The results of immunofluorescent localization studies in the UROtsa cell lines showed that ZIP8 had a punctate pattern that extended throughout the cytoplasm, consistent with the ER, with a concentration in the paranuclear region of the majority of the cells (Figure 7). The intracellular localization of ZIP8 was similar between the parent (Figure 7A, D, G) and As^+3^ (Figure 7B, E, F) or Cd^+2^ (Figure 7C, F, I) transformed UROtsa cell lines although the transformed cells were more likely to have at least some apical localization of ZIP8 in addition to the strong paranuclear localization. These staining patterns are consistent with the staining that was seen in the HPT cells.

**Figure 8:**
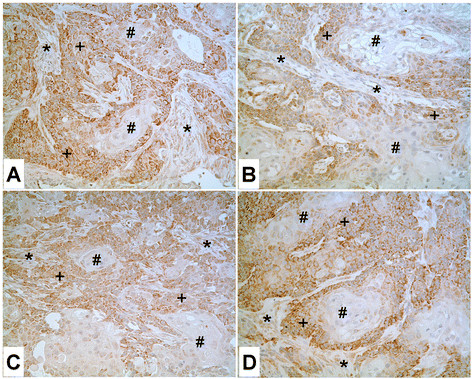
**Immunoperoxidase staining of ZIP8 in tumor heterotransplants.A.** UT As#1. **B.** UT As# 5. **C.** UT Cd#1. **D.** UT Cd#7. The well-differentiated cells in the center of tumor nests show no staining or very weak staining of ZIP8 (#), while the peripheral less differentiated tumor cells show moderate to strong staining for ZIP8 (+). A few spindled shaped stromal cells between the tumor nests show weak staining for ZIP8 (*).

Real-time PCR and western analysis was also employed to determine ZIP8 mRNA and protein expression in extracts prepared from subcuteneous tumor transplants produced in immune compromised mice from each of the Cd^+2^ and As^+3^ transformed cell lines (Figure 6F, G, H). This analysis demonstrated that all the tumor transplants expressed ZIP8 mRNA and the 49 kDa ZIP8 protein. None of the tumor transplants were shown to express the 80 kDa band associated with the glycosylated form of ZIP8. Immunohistochemistry was employed to examine the expression of ZIP8 in the tumor transplants. The results showed that the staining pattern was similar between and among the tumor transplants generated from the Cd^+2^ and As^+3^ transformed cell lines (Figure 8A-D). In all the tumors, the well-differentiated urothelial cells in the center of the tumor nests showed absent or very weak staining for ZIP8 while the peripheral less differentiated tumor cells showed moderate to strong staining in the cytoplasm. There was no evidence of paranulear staining of ZIP8 in any of the tumor transplants. Some spindle shaped stromal cells between the tumor nests also stained weakly for ZIP8.

## Discussion

The first goal of the present study was to determine the expression and localization of ZIP8 in HPT cells. These cells were chosen for analysis since the *in situ* expression of ZIP8 has previously been shown for this cell type along with an association of ZIP8 with Cd-induced damage to the proximal tubule [29-33]. In addition, the renal MDCK cell line, which retains the property of vectorial active transport, has been used to characterize the localization and expression of ZIP8 [30-32]. An analysis of the expression of ZIP8 in the HPT cells largely confirmed what has been found in previous studies employing the MDCK cell line [30-32]. The HPT cells were shown to express two forms of the ZIP8 protein, one at approximately 49 kDa and the other at approximately 80 kDa. The 49 kDa band identified by the ZIP8 antibody is in agreement with the molecular weight expected for the non-glycosylated ZIP8 protein as derived from the sequence available from the NCBI database. The human 49 kDa band identified as ZIP8 in the HPT cells is also in general agreement with that obtained for MDCK cells transfected with the mouse ZIP8 sequence [31]. The approximate 80 kDa band found in extracts of the HPT cells is assumed to be the glycosylated form of the ZIP8 protein. This is based on the molecular weight and association with the membrane fraction of the HPT cell extracts, findings similar to that found for the MDCK cells transfected with the mouse ZIP8 sequence [31]. The ZIP8 protein was also localized to the endoplasmic reticulum and apical cell surface of the HPT cells, an identical localization to that found for the ZIP8-transfected MDCK cells [31]. The finding that there were occasional profiles of HPT cells with paranuclear staining of ZIP8 defines a difference in ZIP8 localization compared to the MDCK cells. There are also findings in other organs and cell types, such as lung and breast epithelium, that show different localizations of ZIP8 and higher molecular weights for the glycosylated form of ZIP8 [34,35]. The significance of glycosylated and non-glycosylated forms is interpreted to reflect processing of the ZIP8 protein for deployment to the plasma membrane. Glycosylation occurs on asparagines (N-linked) initially in the ER. There are no consensus O-linked glycosylation sites in the protein. Final processing of N-linked glycosyl groups on proteins occurs in the Goli apparatus [36,37]. Confocal localization suggests that the protein has considerable ER distribution, and this would be consistent with the 49 kDa form. It is possible that when the initial glycosylation begins, the N-linked glycosyl groups are not enough to significantly increase the molecular weight of the protein and/or to retard the mobility of the protein on a Western blot. The 43 kDa band, corresponding to isoform C which has the first 67 amino acids missing, part of which is the signal peptide, and is predicted not to be transported into the lumen of the ER for glycosylation.

The results of an analysis of ZIP8 in protein extracts of human renal tissue were similar to that of the HPT cells, showing both a 49 kDa and 80 kDa band that was reactive with the ZIP8 antibody. There was also an additional, approximately 43 kDa band in the human renal tissue extract that was reactive with the ZIP8 antibody. This band could be a degradation product due to the processing interval between surgical removal and tissue procurement or it could be an additional isoform of the ZIP8 protein that has a predicted molecular weight 43.1 kDa by NCBI and identified as ZIP8 isoform 2 on the Swiss-Prot database. This 43.1 kDa band was also found in extracts of normal urothelial tissue. The 43 kDa band was not found in extracts of HPT cells or the parental UROtsa cell line. Previous studies have shown ZIP8 to be expressed only in the proximal tubules of the kidney in mice [30]. However, immuno-staining of ZIP8 on archival specimens of human kidney showed ZIP8 to be present in both proximal and distal tubule cells and in some stromal elements in normal urothelium, presenting the possibility of isoform 2 being present in other tubule segments and/or stromal cell types. This discrepancy between mice and human expression patterns could be due to specie specific differences. Overall, the results in the HPT cells regarding the expression of ZIP8 were largely those expected from previous studies. This is important due to the implication of ZIP8 in enhanced cadmium-induced renal proximal tubular damage in mice [30]. The HPT cells have been used as a model for the study of Cd-induced toxicity in the past [38-41] and the current observation that they have basal expression of ZIP8 should provide the research community with an effective *in vitro* model to further elucidate the role of ZIP8 in Cd-induced proximal tubule renal damage.

The second goal of the present study was to determine if ZIP8 was expressed in normal human urothelium and if expression was altered in human urothelial cancer. The results demonstrated that ZIP8 was expressed in the normal urothelium. Immunostaining showed that ZIP8 was expressed in the urothelial cells of all 5 independent specimens of normal urothelium. However, the expression of ZIP8, while uniform within each specimen, was highly variable among the five samples, with staining for ZIP8 varying from very weak to strong in intensity. Immunostaining also showed ZIP8 to have a paranuclear localization in addition to punctate staining within the cytoplasm. Western analysis of ZIP8 expression in 5 independent specimens of normal urothelium showed the presence of the 49 kDa band, but not the higher molecular weight band associated with the glycosylated form of the ZIP8 protein. The corresponding analysis of ZIP8 expression in the UROtsa cell line is of interest regarding the variability of expression and the paranuclear localization of ZIP8 in the normal urothelium. First, the level of expression of the ZIP8 protein in the UROtsa cell line was shown to be dependent on the time following replenishment of the growth medium, with expression being elevated significantly following feeding of the cells with fresh growth medium, followed by a rapid reduction in expression within 36 hrs of the addition of fresh growth medium. It has also been shown that the availability of Zn^+2^ can influence the trafficking of the ZIP8 protein to the apical cell surface in MDCK cells [32]. One can speculate that the variability of expression of ZIP8 demonstrated among the independent specimens of normal urothelium may reflect differences in the nutritional status of the patient from which the samples originate. The possibility that ZIP8 expression can vary with nutritional status would render interpreting differences in expression between levels in tissues and fluids from normal versus disease states very difficult. Second, the ZIP8 protein was also localized to the paranuclear region of the parental UROtsa cells, a finding in agreement with the paranuclear localization of the ZIP8 protein in the five patient samples of normal urothelium. The association of ZIP8 protein with the cell nucleus could indicate a possible involvement in providing Zn^+2^ to Zn-requiring transcription factors. The present findings appear to be the first description of ZIP8 expression and localization in human urothelium.

As part of the above goal it was also proposed to determine if ZIP8 expression was altered in human urothelial cancer. As noted above, the wide variability of expression of ZIP8 in normal urothelium renders comparison very difficult between the normal and malignant urothelial cells. The most striking finding from the analysis was that out of the 14 cases of low and high grade urothelial cancer examined for ZIP8 expression, there was one high grade, invasive urothelial cancer that showed no expression of the ZIP8 protein. An additional difference in the expression of ZIP8 among the urothelial cancers was that there was no paranuclear localization of ZIP8 in any of the high grade urothelial cancers that showed positive staining for ZIP8. Otherwise, expression in the remaining high and low grade cancers displayed a wide variability of ZIP8 expression similar to that noted in specimens of normal urothelium. The fact that the one urothelial cancer that did not express the ZIP8 protein was in the high grade invasive group could be important since the loss of ZIP8 could be associated with tumor progression. This would need to be confirmed on a much larger sample set of urothelial tumors having data on patient outcome.

The final goal of the study was to determine ZIP8 expression and localization in human urothelial cells transformed by Cd^+2^ and As^+3^. The results showed that both Cd^+2^ and As^+3^ transformed UROtsa cells and their tumor transplants expressed higher levels of ZIP8 mRNA and protein compared to the parental cell line. There was no notable difference in the expression of ZIP8 between the Cd^+2^ and As^+3^ transformed cell lines, ruling out a Cd^+2^ specific alteration in ZIP8 expression that might be associated with the development of urothelial cancer. One difference noted between the As^+3^ and Cd^+2^ transformed cell lines compared to the parental UROtsa cell line was that the transformed lines consistently expressed the 80 kDa band of ZIP8 associated with the glycosylated form of the protein. The parental UROtsa cell line displayed the 80 kDa band only transiently after the cells were fed fresh growth medium. The transformed cell lines also showed some localization of ZIP8 to the cell membrane, but the majority was localized to the cytoplasm and paranuclear region of the cells. Again, these differences are difficult to interpret due to the time dependence of ZIP8 expression with growth medium replenishment in the parental UROtsa cell line. Similar to that found for the archival specimens of high grade urothelial cancer, the tumor transplants generated from the As^+3^ and Cd^+2^ transformed cells showed no evidence of paranuclear staining for ZIP8.

It remains to be elucidated why or how ZIP8 is overexpressed in metal transformed cells. It was unexpected that As^+3^-transformed cells also over-express this transporter. As^+3^ is not expected to be transported by ZIP8 due to the divergent properties of these metals. As^+3^ exists as a trihydroxylated, neutral species known as arsenous acid As(OH)_3_ with the pK for the donation of the first hydrogen being greater at pH 9.0 [42], and is thought to be transported via the aquaporin transporters [43,44]. It is well known that global gene expression patterns are considerably altered during metal carcinogenesis, and that alterations in epigenetic regulation have been appreciated to play a fundamental role [45]. Epigenetic alterations leading to the overexpession or silencing of specific loci have been correlated to methylation/demethylation of CpG islands and post-translational modifications of histone tails within the promoters of altered genes. Specific explanations for why particular loci are silenced or conducive to overexpression by long-term exposure and/or transformation by metals still remain to be determined, although in a few specific cases, alteration in the expression or activity of methylases and demethylases have be identified. Epigenetics alterations are thus suspected in the case of ZIP8 overexpression in Cd^+2^- and As^+3^-transformed cells. It is tempting to speculate that specific metal transport pathways may be involved.

## Conclusions

The study is the first to show that ZIP8 is expressed in normal urothelium. ZIP8 was also shown to be expressed in 13 of 14 urothelial cancers; with one high grade, invasive urothelial cancer being negative for ZIP8 expression. ZIP8 was shown to have a paranuclear localization in normal urothelium, but not in high grade urothelial cancers. The parental UROtsa cell line and its As^+3^ and Cd^+2^ counterparts showed a similar pattern of ZIP8 expression when compared to the normal urothelium and urothelial cancers and should provide a human model system to study ZIP8 expression in bladder disease. The study also shows that the HPT cells should provide an effective human *in vitro* model system for the study of the role of ZIP8 in proximal tubule damage by Cd^+2^ and possibly other heavy metals.

## Methods

### Cell culture

Stock cultures of HPT cells for use in experimental protocols were grown using serum-free conditions in a 37°C, 5% CO_2_:95% air atmosphere as previously described by this laboratory [46,47]. The cells were fed fresh growth medium every 3 days, and at confluence, the cells were subcultured using trypsin-EDTA (0.05%, 0.02%). Stock cultures of the parental UROtsa cell line were maintained in Dulbecco’s modified Eagle’s medium (DMEM) containing 5% v/v fetal calf serum in a 37°C, 5% CO_2_:95% air atmosphere [21]. The isolation and growth of the 7 isolates of the Cd^+2^ transformed UROtsa cells and 6 isolates of the As^+3^ transformed UROtsa cells have been described previously [25-27]. They were grown and maintained using identical conditions. Confluent flasks were sub-cultured at a 1:4 ratio using trypsin-EDTA (0.05%, 0.02%) and the cells were fed fresh growth medium every 3 days.

### Human and tumor transplant tissue for immunohistochemistry, real-time PCR and western analysis of ZIP8 expression

Tissue for the immunohistochemical analysis of ZIP8 expression in human bladder were obtained from archival paraffin blocks that originated from previously completed patient diagnostic procedures. These archival specimens contained no patient identifiers and use was approved by the University of North Dakota Internal Review Board. Fresh and paraffin-embedded, formalin-fixed tumor samples originating from the As^+3^ and Cd^+2^ transformed UROtsa cell lines were pre-existing specimens from previous studies [21,25,26]. Human kidney and urothelial tissue used for real-time PCR analysis and western analysis of ZIP8 mRNA and protein expression were obtained as medical waste from surgical specimens following completion of all diagnostic protocols. These specimens contained no patient identifiers and use was approved by the University of North Dakota Internal Review Board.

### Expression of ZIP8 mRNA and protein in tissue and cell culture preparations

The preparation of total RNA and protein from cultured cells and tissues has been described previously [21,25,26]. For the isolation of cytosolic and membrane associated proteins, the tissue was snap frozen in liquid nitrogen and was ground to a fine powder. Proteins were extracted from the powdered tissue by dissolving it in T-PER reagent (Thermo Scientific, Rockford, IL) and the DNA was sheared by passing the tissue extract through a 23-gauge needle. The tissue extract was centrifuged at 16,000 × g for 20 minutes at 4°C. Isolation of the membrane and cytosolic fractions was carried out by centrifuging the supernatant at 100,000 g for 30 minutes at 4°C in a TLA-100.3 rotator (Beckman TL100) ultracentrifuge. The clear red supernatant representing the cytosolic fraction was decanted. The light-brown pellet representing the membrane fraction was rinsed and resuspended in T-PER reagent. Protein concentration was determined by the bicinchoninic acid (BCA) protein assay (Pierce Chemical Co., Rockford, IL) before 100 mM dithiothreitol (DTT) was added to each sample.

The expression of ZIP8 mRNA was determined using real-time RT-PCR and ZIP8 specific primers were obtained from Qiagen (Valencia, CA). Briefly, 1 μg of purified RNA was subjected to complementary DNA (cDNA) synthesis using the iScript cDNA synthesis kit (Bio-Rad Laboratories, Hercules, CA) in a total volume of 20 μL. Real-time PCR was performed utilizing the SYBR Green kit (Bio-Rad Laboratories) with 2 μL of cDNA, 0.2 μM primers in a total volume of 20 μL in an iCycler iQ real-time detection system (Bio-Rad Laboratories). Amplification was monitored by SYBR Green fluorescence. The level of ZIP8 mRNA was determined relative to that of UROtsa cells grown in serum-containing medium using serial dilutions of this sample as the standard curve. The resulting relative levels were then normalized to the fold change in β-actin expression assessed by the same assay using the primers, sense: CGACAACGGCTCCGGCATGT and antisense: TGCCGTGCTCGATGGGGTACT, giving a product size of 194 base pairs and with the cycling parameters of annealing/extension at 62°C for 45 seconds and denaturation at 95°C for 15 seconds.

The expression of ZIP8 protein was determined by western blotting. Briefly, protein samples (20 μg) were separated on a 12.5% sodium dodecyl sulfate (SDS)-polyacrylamide gel and transferred to a hybond-P polyvinylidene difluoride membrane (Amersham Biosciences, Piscataway, NJ). Membranes were blocked in Tris-buffered saline (TBS) containing 0.1% Tween-20 (TBS-T) and 5% (wt/vol) nonfat dry milk for 1 hr at room temperature. After blocking, the membranes were probed with the ZIP8 primary antibody (0.18 μg/ml) in blocking buffer for 1 hr at room temperature. The primary antibody for the monitoring of ZIP8 expression was an affinity purified rabbit polyclonal antibody produced by Open Systems, Inc (Huntsville, AL) using the peptide sequence QNGHTHFGNDNFGPQEKTH previously described in the literature to generate an antibody specific for ZIP8 [34]. After washing 3 times with TBS-T, the membranes were incubated with the anti-rabbit secondary antibody (1:2000) in antibody dilution buffer for 1 hour. The blots were visualized using the Phototope-HRP (horseradish peroxidase) Western blot detection system (Cell Signaling Technology, Beverly, MA).

### Immunohistochemical analysis of ZIP8 expression

Tissues were routinely fixed in 10% neutral buffered formalin for 16–18 hours. All tissues were transferred to 70% ethanol and dehydrated in 100% ethanol. Dehydrated tissues were cleared in xylene, infiltrated, and embedded in paraffin. Tissue sections were cut at 3–5 μm for use in immunohistochemical protocols. Prior to immunostaining, sections were immersed in preheated citrate buffer pH 6.0 and heated in a steamer for 20 minutes. The sections were allowed to cool to room temperature and immersed into TBS-T (Dako, Carpinteria, CA) for 5 minutes. The ZIP8 antibody was used at 0.45 μg/ml. Liquid diaminobenzidine was used for visualization. Slides were rinsed in distilled water, dehydrated in graded ethanol, cleared in xylene, and coverslipped.

### Immunolocalization of ZIP8 in human proximal tubule and UROtsa cells

The cells were grown in 24 well plates containing 12 mm glass coverslips at 37°C, 5% CO_2_. Cells at confluent density were then fixed and stained using previously described procedures [25,26]. Briefly, cells were fixed in 3.7% buffered, methanol-free formaldehyde (Polysciences, Inc, Warrington, PA) for 15–20 minutes at room temperature. Coverslips were then quenched of free aldehyde with 0.1 M NH_4_Cl for 15 minutes, followed by permeabilization with 0.1% Saponin for 10 minutes. Cells were stained for ZIP8 by incubation for 45 minutes at room temperature with 9.0 μg/ml ZIP8 antibody. The ZIP8 primary antibody was detected by incubating cells with 2.7 μg/ml of Alexa-Fluor 488 goat anti-rabbit antibody (Invitrogen, Carlsbad, CA) for 45 minutes at room temperature. Controls consisted of coverslips treated with the secondary antibody only. All controls stained appropriately and had virtually no staining when photographed under the same settings that were used for experimental cells. For experiments determining the localization of ZIP8 near the nucleus, staining was carried out as indicated above followed by staining with a 5 μM solution of To-PRO-3 iodide (Invitrogen) for 45 minutes at room temperature. All coverslips were mounted in ProLong Gold anti-fade reagent with 4′,6-diamidino-2-phenylindole (DAPI) (Invitrogen) for nuclear counter staining. Cells were observed and images captured using a Zeiss LSM 510 Meta Confocal Microscope with LSM 510 software (Carl Zeiss MicroImaging Inc). Images were obtained by capturing z-slices at a depth of 0.5 μm. DAPI images of the same fields were captured by epifluorescence.

## Abbreviations

As+3 = Arsenite; Cd+2 = Cadmium; DMEM = Dulbecco’s modified Eagle’s medium; BCA = Bicinchoninic acid; DTT = Dithiothreitol; cDNA = Complementary DNA; ER = Endoplasmic reticulum; Hr = Hour; HPT = Human proximal tubule; SDS = Sodium dodecyl sulfate; TBS = Tris-buffered saline; TBS-T = Tris-buffered saline containing 0.1% Tween-20; HRP = Horseradish peroxidase; DAPI = 4′,6-diamidino-2-phenylindole.

## Competing interests

None of the authors’ have competing interests.

## Authors’ contributions

AA: Postdoctoral fellow. RT-PCR and western analysis of ZIP8 expression. TB: Undergraduate research summer student who validated the initial finding of elevated ZIP8 mRNA expression from microarray data. SG: Supervised the postdoctoral fellow in western analysis and assisted with data analysis. MAS & ZDZ: Procurement of human and animal tissue, mouse autopsy, tissue fixation, and evaluation of immunostaining and histology. JD: Supervised, trained, and assisted postdoctoral fellow with immunofluorescent localization. DAS: Worked on the manuscript with postdoctoral fellow, designed study, final data integration. SS: Wrote the manuscript and trained and supervised postdoctoral fellow in cell culture and RT-PCR analysis. All authors read and approved the final manuscript.
